# IL-34 attenuates acute T cell-mediated rejection following renal transplantation by upregulating M2 macrophages polarization

**DOI:** 10.1016/j.heliyon.2024.e24028

**Published:** 2024-01-03

**Authors:** Bin Ni, Dongliang Zhang, Hai Zhou, Ming Zheng, Zijie Wang, Jun Tao, Zhijian Han, Xiaobin Ju, Ruoyun Tan, Min Gu

**Affiliations:** a Department of Urology, the Second Affiliated Hospital of Nanjing Medical University, 121# Jiangjiayuan Road, Nanjing, Jiangsu, China; b Department of Urology, the First Affiliated Hospital of Nanjing Medical University, 300# Guangzhou Road, Nanjing, Jiangsu, China

**Keywords:** Kidney transplantation, T cell-mediated rejection, IL-34, M2 macrophage

## Abstract

**Objective:**

To investigate the role of Interleukin-34 (IL-34) in acute T cell-mediated rejection (TCMR) following renal transplantation.

**Methods:**

The mice acute TCMR model of renal transplantation was established and identified by hematoxylin and eosin (HE) and immunohistochemistry (IHC) staining. Then, IHC staining of IL-34 was also performed to determine the expression of IL-34 in allografts. Recipients were infected with IL-34 overexpression adeno-associated virus, infection efficiency of which was estimated by enzyme linked immunosorbent assay (ELISA), Western blot, and immunofluorescence. HE and IHC staining were used to estimate the grades of TCMR. Flow cytometry was performed on lymphocytes in spleens of recipients including regulatory T cells (Tregs) and M2 macrophages. The expression of cytokines in vivo was analyzed by Mouse Cytokine Grp I Panel. Finally, Tregs and M2 macrophages were cultured *in vitro* and treated with IL-34 to observe the effects of IL-34 on the differentiation of the cells.

**Results:**

The mouse TCMR model was successfully established by HE, periodic acid shiff (PAS), CD4 and CD8 IHC staining. The expression of IL-34 was significantly decreased in allografts with TCMR. BALB/c mice were successfully infected with IL-34 overexpression adeno-associated virus. Subsequently, the grade of rejection in mice TCMR model was evaluated by HE and IHC staining according to Banff criteria. It is suggested that the grade of TCMR in IL-34 overexpressed mice was significantly decreased. IHC staining and Flow cytometry showed that the proportion of Tregs and M2 macrophages in the spleens and allografts were significantly increased in IL-34 overexpressed mice. Serum levels of interferon-gamma (IFN-γ), IL-17 and tumor necrosis factor-alpha (TNF-α) were downregulated in IL-34 overexpressed mice. Moreover, IL-34 could promote macrophage M2 polarization, while failed to promote differentiation of naïve T cells into Tregs *in vitro*.

**Conclusion:**

Overexpression of IL-34 may attenuate the progression of TCMR episodes in allografts by increasing the polarization of M2 macrophages in the spleens and allografts, which may become a potential therapeutic strategy for TCMR.

## Abbreviations

AAV: adeno-associated virus
BMDMs: bone-marrow-derived macrophages
CSF-1: colony-stimulating factor-1
ELISA: enzyme linked immunosorbent assay
HE: hematoxylin and eosin
IHC: immunohistochemistry
IL-34: interleukin-34
PAS: periodic acid shiff
qRT-PCR: quantitative real-time polymerase chain reaction
SYN: syngeneic
Tregs: regulatory T cells
TCMR: T cell-mediated rejection
IFN-γ: interferon-gamma
TNF-α: tumor necrosis factor-alpha

## 1 Introduction

Patients with uremia need long-term renal replacement therapy and experience the effects of various ailments for a long time. Renal transplantation has greatly improved the quality of life of these patients. In the early stage of development in the field of renal transplantation, postoperative acute T cell mediated rejection (TCMR) is a focus of clinical attention. Subsequent advancements in immunosuppression have substantially diminished the rates and severity of clinically manifested TCMR in kidney transplant recipients, reaching 5%–15 % in the first year [1]. Although the incidence of TCMR has significantly decreased, there are still many problems to be solved. It is reported that chronic graft injury and loss of function are significantly associated with recurrent or persistent TCMR [2]. Besides, the concept of chronic active TCMR was proposed at the 2017 Banff Conference. Various indications illustrate that TCMR still has a significant impact on the long-term prognosis of allografts at present, and the mechanism of occurrence and development remains to be further studied.

Interleukin-34 (IL-34) is a cytokine first reported in 2008 [3]. The human IL-34 gene is located on chromosome 16q22.1 and is made up of 242 amino acids. While IL-34 share the same receptor CD115 with colony-stimulating factor-1 (CSF-1), they lack homology in the nucleic acid sequence [4,5]. Notably, IL-34 exhibits higher affinity to CSF-1R than CSF-1, and their binding modes differ. So far, it is reported that IL-34 is secreted by a variety of cells and involved in the differentiation and survival of macrophages, monocytes and dendritic cells, as well as the development of microglia and Langerhans cells [6,7]. Due to its close association with mononuclear macrophages, the immunomodulatory function of IL-34 has been highly anticipated [8]. It has been found that IL-34 induced in vivo and *in vitro* regulatory T cells (Tregs) through monocytes polarization toward M2 macrophages to protect allografts from acute and chronic rejection. Bézie first proposed that as an inhibitory Treg cell-specific cytokine, IL-34 could mediate immune tolerance by inhibiting donor-specific immunity [9]. The above results suggest that IL-34 plays a certain role in inducing and maintaining immune tolerance after organ transplantation. However, the specific role and related mechanisms of IL-34 in the induction of immune tolerance after renal transplantation remain unexplored. Thus, we hypothesize that IL-34 could induce the differentiation of Tregs and M2 macrophages, thereby inhibiting acute TCMR of allografts and inducing immune tolerance.

Our study found that the expression of IL-34 decreased in the allografts with TCMR. Overexpression of IL-34 increased the expression of Tregs and M2 macrophages in the spleens and allografts of TMCR mice. IL-34 promoted macrophage M2 polarization but not the differentiation of Tregs *in vitro*.

## 2 Materials and methods

### 2.1 Mice

Male C57BL/6 (H-2^b^) and BALB/c (H-2^d^) mice, weighing 20–25 g, were obtained from Nanjing Medical University. All mice were housed in groups of five per cage in a room with a 12-h light/dark cycle, maintained at a temperature of 20–26 °C with a relative humidity of 40–60 %. All the experimental procedures were approved by the Institutional Animal Care and Use Committee at Nanjing Medical University (IACUC-2109025).

### 2.2 Kidney transplantation

Kidney transplantation was carried out on BALB/c mice, who received a kidney from a C57BL/6 donor as an allograft or from a BALB/c donor as a syngeneic (SYN) graft. Ectopic kidney transplantation was performed as described previously [10]. In brief, the left donor kidney was transplanted to the abdominal of the recipient with both native kidneys of the recipient retained and artery and vein were anastomosed to the recipient's abdominal aorta and vena cava using 10-0 sutures. The donor ureter was embedded into the recipient's bladder. The technical success rate of the transplantation procedure was 90 %.

HBAAV2/9-m-il34-3 × flag-Zsgreen (AAV-IL-34) and HBAAV2/9-Zsgreen (AAV-Con) were constructed and packaged by HANBIO Company. The AAV-IL-34 and AAV-Con were injected via the tail vein (1.8*10^11^ v.g for each mouse). Further details are available on request. Mice were divided to the following groups: SYN-AAV-Con, TCMR-AAV-Con, SYN-AAV-IL-34 and TCMR-AAV-IL-34.

### 2.3 Cell culture and treatment

Bone-marrow-derived macrophages (BMDMs) were isolated as previously described [11]. Briefly, bone marrow suspension was obtained by flushing the bone marrow cavity of the femur and tibia. Monocytes were isolated from the bone marrow suspension using Lymphoprep (MLSM1092, MULTISCIENCES Biotech). BMDMs were cultured in Dulbecco's modified Eagle's medium (DMEM) containing 10 % (v/v) FBS (cat: 04-001-1ACS, BIOIND), 1 % (v/v) antibiotics (cat: 15070063, Gibco) and 30 ng/ml mouse M-CSF (cat: CB34; Novoprotein Scientific Inc, Suzhou, CHINA). The culture medium was refreshed every 3 days. On day 7, BMDMs were treated with varying concentrations of interleukin-4 (IL-4) at 10, 20, and 40 ng/μl.

### 2.4 Western blot assay

As previously described in Ref. [12], total protein was extracted by RIPA lysis buffer (Beyotime, Shanghai) and the concentrations were measured with BCA assay (Beyotime, Shanghai). The primary antibodies were IL-34 (cat: PA5-95624, Invitrogen, 1:1000), CD206 (cat: 60143-1-lg, Proteintech, 1:1000) and GADPH (1:20000). Following incubation with horseradish peroxidase-conjugated secondary antibodies (1:2000, Proteintech), the signal was detected with an imaging system.

### 2.5 Histology and immunohistochemistry (IHC) staining

The kidney grafts were obtained and processed with 4 % paraformaldehyde fixation and paraffin embedment. 5 μm thickness sections were stained with hematoxylin and eosin (HE) and periodic acid–Schiff (PAS), according to the standard steps. The sections were immune-stained with antibodies against CD4 (cat: ab183685, Abcam, 1:800), CD8 (cat: ab209775, Abcam, 1:800), FOXP3 (cat: ab215206, Abcam, 1:400), IL-34 (cat: PA5-95624, Invitrogen, 1:400) and CD206 (cat: 60143-1-lg, Proteintech, 1:400). The sections were incubated with primary antibodies at 4 °C overnight and secondary antibody for 1 h at room temperature, subsequently subjected to DAB staining. Sections were viewed and number of positive area was determined.

### 2.6 Quantitative real-time polymerase chain reaction (qRT-PCR)

Total RNA was extracted using the RNA Isolation Kit (RC112-01, Vazyme) and the extracted RNA was quantified and reverse transcribed using HiScript III All-in-one RT SuperMix Perfect for qPCR kit (R333-01, Vazyme) and gene expression was determined by qRT-PCR (Q341-AA, Vazyme). Primer sequences were list in Table 1.

**Table 1:** Primer sequences.

Gene	Primer sequences (5′-3′)
IL-34	F: CTGGCTGTCCTCTACCCTGA
	R: TGTCGTGGCAAGATATGGCAA
CD206	F: CTCTGTTCAGCTATTGGACGC
	R: CGGAATTTCTGGGATTCAGCTTC
FOXP3	F: CCCATCCCCAGGAGTCTTG
	R: ACCATGACTAGGGGCACTGTA
CD80	F: CTGCAAAGGACTTCAGAAACCT
	R: AGGCTTCACCTAGAGAACCGT
iNOS	F: GTTCTCAGCCCAACAATACAAGA
	R: GTGGACGGGTCGATGTCAC
CD86	F: TGTTTCCGTGGAGACGCAAG
	R: TTGAGCCTTTGTAAATGGGCA
GAPDH	F: GAAGGTCGGTGTGAACGGAT
	R: CCCATTTGATGTTAGCGGGAT

### 2.7 IL-34 ELISA assay

Serum IL-34 concentration was detected with the Mouse IL-34 ELISA kit (cat: CSB-EL011657MO, CUSABIO) in accordance with the manufacturer's instructions.

### 2.8 Flow cytometry

The harvested spleen was grinded and filtered through 40-μm mesh to get single-cell suspension. The following fluorochrome-conjugated antibodies were used for cell staining: CD3 (cat: 45-0031, eBioscience), CD4 (cat: 53-0041, eBioscience), CD8 (cat: 12–0081, eBioscience), CD25 (cat:62–0251, eBioscience), FOXP3 (cat: 17–5773, eBioscience), F4/80 (cat: 12–4801, eBioscience), CD206 (cat:17–2061, eBioscience) and CD62 (cat: 17–0621, eBioscience). Single-cell suspensions were analyzed by an CytoFLEX flow cytometer (Beckman Coulter), and the obtained data were subjected to analyses using CytExpert software.

### 2.9 Luminex liquid suspension chip

Luminex liquid suspension chip analyses was performed to detect the concentrations of serum interferon-gamma (IFN-γ), IL-17 and tumor necrosis factor-alpha (TNF-α) by Wayne Biotechnologies (Shanghai, China). The Bio-Plex Pro Mouse Cytokine Grp I Panel 23-plex was applied according to the manufacturer's instructions.

### 2.10 Statistical analyses

All data are shown as mean ± SD. All the experiments were biological replicated five times and technical replicated three times. Unpaired student's t-test was used to analyze the differences between the groups using GraphPad Prism 9. A value of P < 0.05 was considered statistically significant.

## 3 Results

### 3.1 Construction and characterization of the mouse model of TCMR

**Fig. 1:**
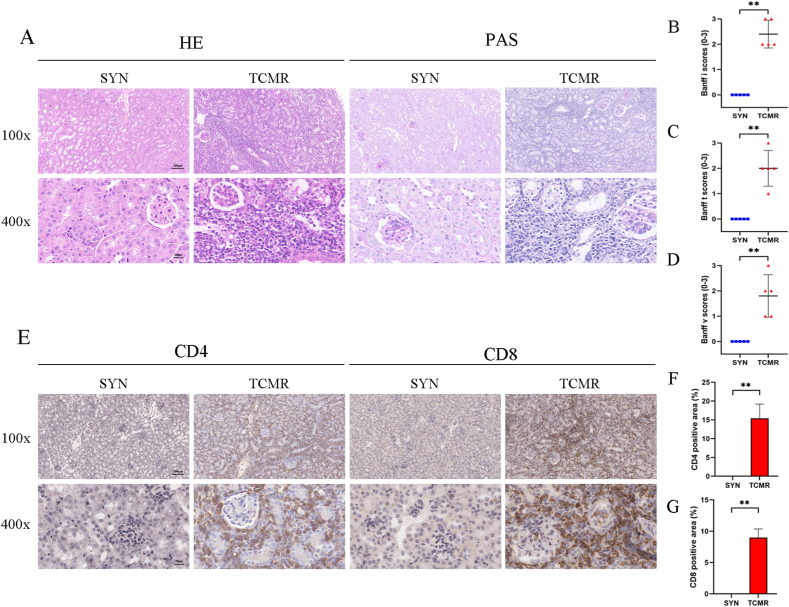
Construction and validation of allogeneic mice renal transplant TCMR model. (A) Representative micrographs for hematoxylin and eosin (HE) and periodic acid–Schiff (PAS) staining in grafts (magnification: 100×, scale bar = 100 μm; magnification: 400×, scale bar = 20 μm). (B–D) Graphic presentation showing the i (B), t (C) and v (D) scores according to Banff 2019. (E) Representative staining images for CD4 and CD8 immunohistochemistry (IHC) staining in grafts (magnification: 100×, scale bar = 100 μm; magnification: 400×, scale bar = 20 μm) (F–G) Graphic presentation showing the CD4 (F) positive area and CD8 (G) positive area in grafts among groups. *P < 0.05, **P < 0.01. Data are presented as means ± SD.

To explore the role of IL-34 in TCMR after renal transplantation, we constructed and validated a mouse renal transplant TCMR model. HE and PAS showed that the morphology and structure of glomeruli and tubules in the SYN group were basically normal, and a small amount of tubular dilatation and cellular edema were partially observed, which was consistent with ischemia-reperfusion injury and surgery-induced acute renal injury manifestations. However, diffuse mononuclear cell infiltration in the interstitial and mononuclear cell infiltration with expansion and brush edge shedding in the renal tubules were observed in TCMR allografts, which was consistent with the pathologic manifestations of TCMR (Fig. 1A). According to the Banff2019 TCMR diagnostic criteria [13], renal interstitial inflammation (i), tubulitis (t), and intrarenal arteritis (v) were scored. The results showed that the scores in the TCMR group were significantly higher than those in the SYN group (*P* < 0.01) (Fig. 1B–D). IHC staining showed remarkable infiltration of CD4^+^ and CD8^+^ T cells infiltration in the TCMR allografts (*P* < 0.01) (Fig. 1E–G). Taken together, allogeneic mice renal transplant TCMR model was successfully established and validated.

### 3.2 IL-34 was decreased in TCMR allografts

**Fig. 2:**
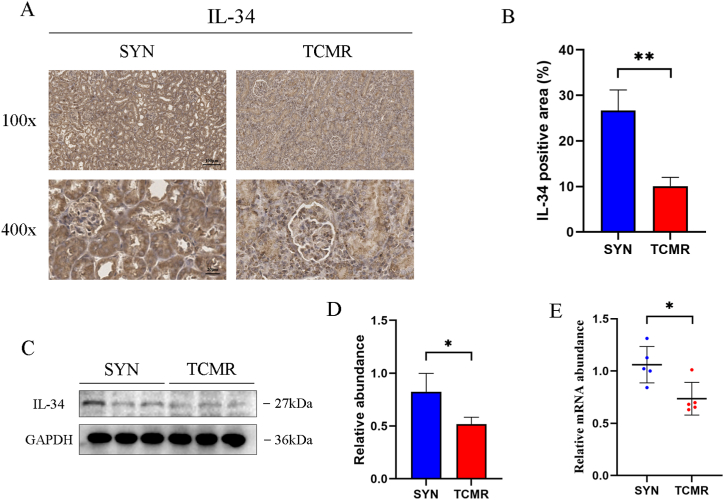
IL-34 expression was decreased in TCMR allografts. (A) Representative immunohistochemistry (IHC) staining images of interleukin-34 (IL-34) in grafts (magnification: 100×, scale bar = 100 μm; magnification: 400×, scale bar = 20 μm) (B) Graphic presentation showing the quantification of IL-34 positive area in grafts. (C, D) Western blot (C) and quantitative analysis (D) showing the protein abundance of IL-34 in grafts. (E) Quantitative real-time polymerase chain reaction (qRT-PCR) analysis showing the mRNA of IL-34 in grafts. *P < 0.05, **P < 0.01. Data are presented as means ± SD.

To clarify the role and mechanism of IL-34 in TCMR allografts, expression of IL-34 in allografts of SYN group and TCMR group was explored. As shown in Fig. 2A–B, IL-34 was widely expressed in the tubular epithelium of SYN group, whereas the expression of IL-34 in the tubular epithelium of TCMR allografts was significantly reduced (*P* < 0.01). On the contrary, the specific expression of IL-34 appeared in diffusely infiltrated mononuclear cell in the mesenchyme. The protein and mRNA expression of IL-34 were significantly decreased in the TCMR group compared to that in the SYN group (*P* < 0.05) (Fig. 2C–E, S2A). The results indicated that IL-34 was decreased in TCMR allografts.

### 3.3 IL-34 overexpression ameliorated the progress of TCMR

To further clarify the role of IL-34 on the rejection reaction of allografts, adeno-associated virus mediated overexpression of IL-34 (AAV-IL-34) was injected intravenously to intervene recipients and blood was collected to analyze by ELISA through the periorbital region at day 21 after administration (S1A). Compared with wild type mice, there was no significant difference in serum IL-34 concentration in the AAV-control group(*P* > 0.05), whereas it significantly upregulated in AAV-IL-34 mice. The mice were executed at day 7 after transplantation. Serum IL-34 in the TCMR group was significantly higher than that in the SYN group (*P* < 0.01). Notably, serum IL-34 in the TCMR-AAV-IL-34 group was significantly higher than that in the TCMR-AAV-control group (*P* < 0.01) (S1B). In addition, IL-34 protein abundance in liver tissue of TCMR-AAV-IL-34 group was significantly higher than TCMR-AAV-control group (*P* < 0.01) (S1C, D).

**Fig. 3:**
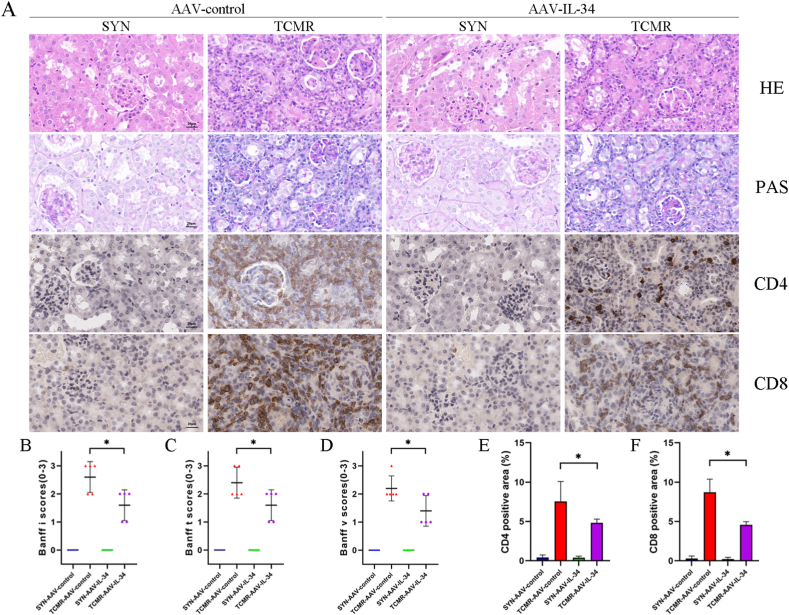
IL-34 overexpression ameliorated the progress of TCMR. (A) Representative micrographs for hematoxylin and eosin (HE), periodic acid–Schiff (PAS), CD4 and CD8 staining in grafts (scale bar = 20 μm). (B–D) Graphic presentation showing the i (B), t (C) and v (D) scores in grafts among groups as indicated. (E, F) Graphic presentation showing the CD4 positive area (E) and CD8 positive area (F) in grafts among groups as indicated. *P < 0.05, **P < 0.01. Data are presented as means ± SD.

As shown in Fig. 3A, HE and PAS staining indicated cortical interstitial inflammatory cell infiltration, tubulitis and arteritis in TCMR-AAV-control allografts, which were markedly attenuated in the TCMR-AAV-IL-34 allografts. IHC staining suggested CD4 and CD8 protein abundance was largely reduced in the TCMR-AAV-IL-34 allografts compared to those in the TCMR-AAV-control allografts. According to Banff2019, i, t and v scores significantly decreased in the TCMR-AAV-IL-34 allografts (*P* < 0.05) (Fig. 3B–D). Similarly, there was a decrease in the infiltration of CD4^+^ and CD8^+^ T cells in the TCMR-AAV-IL-34 allografts (*P* < 0.05) (Fig. 3E and F). The results indicated that IL-34 overexpression ameliorated the progress of TCMR.

### 3.4 IL-34 overexpression protected grafts by upregulating differentiation of Tregs and M2 macrophages

**Fig. 4:**
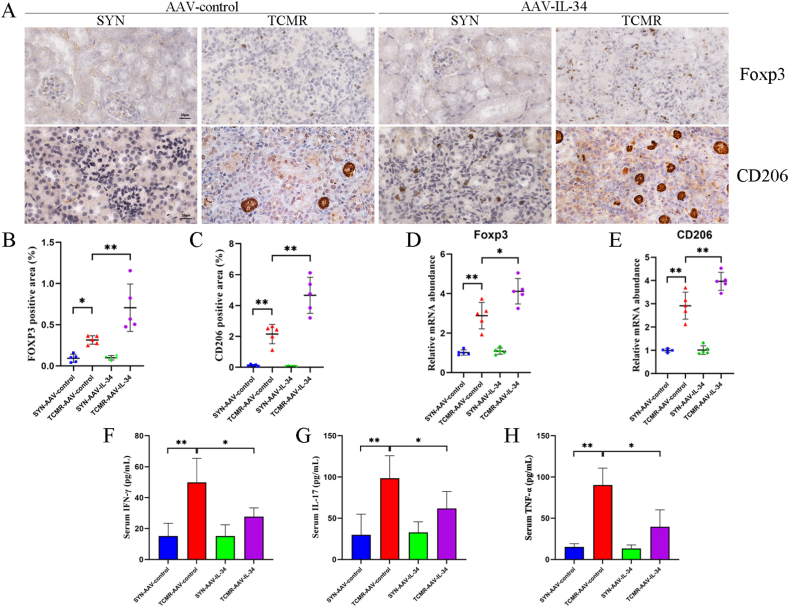
IL-34 overexpression protected grafts by upregulating regulatory T cells (Tregs) and M2 macrophages and inhibiting pro-inflammatory cytokines. (A) Representative immunohistochemistry (IHC) staining for Foxp3 and CD206 in grafts (scale bar = 20 μm). (B–C) Foxp3 positive cells (B) and CD206 positive cells (C) in grafts among groups as indicated. (D, E) Quantitative real-time polymerase chain reaction (qRT-PCR) analysis showing the mRNA abundance of Foxp3 (D) and CD206 (E) in grafts. (F–H) Graphic presentation showing the concentrations of serum interferon-gamma (IFN-γ), IL-17 and tumor necrosis factor-alpha (TNF-α) among groups. *P < 0.05, **P < 0.01. Data are presented as means ± SD.

It has been reported that Tregs and M2 macrophages were involved in immunological effects of IL-34 in the rat heart transplantation model [9]. Hence, the distribution of Tregs and M2 macrophages in allografts were detected. Foxp3 and CD206 positive staining significantly increased in the TCMR-AAV-IL-34 allografts compared to the TCMR-AAV-control allografts (*P* < 0.01) (Fig. 4A–C). The mRNA abundance of Foxp3 (*P* < 0.05) and CD206 (*P* < 0.01) was significantly upregulated in the TCMR-AAV-IL-34 allografts (Fig. 4D and E). In addition, the concentrations of serum IFN-γ, IL-17 and TNF-α in the TCMR-AAV-IL-34 group were significantly lower than those in the TCMR-AAV-control group (*P* < 0.05), which suggested that IL-34 overexpression inhibited the secretion of pro-inflammatory cytokines (Fig. 4F–H).

### 3.5 IL-34 overexpression increased the expression of regulatory T cells (Tregs) and M2 macrophages in the spleens of TMCR mice

**Fig. 5:**
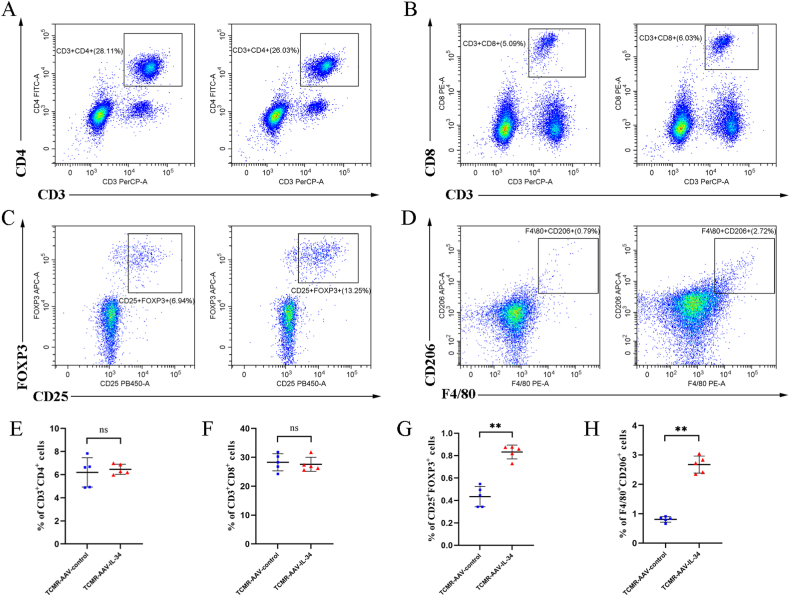
IL-34 overexpression increased the expression of regulatory T cells (Tregs) and M2 macrophages in the spleens of TMCR mice. (A–D) Representative plots of splenic CD3^+^CD4^+^ T cells (A), CD3^+^CD8^+^ T cells (B), CD4^+^CD25^+^ FOXP3^+^ Tregs (C) and F4/80^+^CD206^+^ macrophages (D) analyzed by flow cytometry. (E–H) Graphic presentation showing the percentages of splenic CD3^+^CD4^+^ T cells (E), CD3^+^CD8^+^ T cells (F), CD4^+^CD25^+^ FOXP3^+^ Tregs (G) and F4/80^+^CD206^+^ macrophages (H). *P < 0.05, **P < 0.01. Data are presented as means ± SD.

To further clarify the changes of circulating lymphocytes in recipients after renal transplantation, spleens of recipient were subjected to flow cytometry. No statistically significant difference was observed between CD3^+^CD4^+^ and CD3^+^CD8^+^ T cells in the TCMR-AAV-control group and the TCMR-AAV-IL-34 group, which suggested that IL-34 could not influence the T-cell subpopulation (*P* > 0.05) (Fig. 5A–B, E-F). We further found that CD4^+^CD25^+^ FOXP3^+^ Tregs and F4/80^+^CD206^+^ macrophages were significantly higher in the TCMR-AAV-IL-34 group than in the TCMR-AAV-control group (*P* < 0.01) (Fig. 5C–D, G-H), demonstrating that IL-34 overexpression increased the expression of Tregs and M2 macrophages and promoted their infiltration into the transplanted kidney.

### 3.6 IL-34 promoted macrophage M2 polarization but not the differentiation of Tregs *in vitro*

**Fig. 6:**
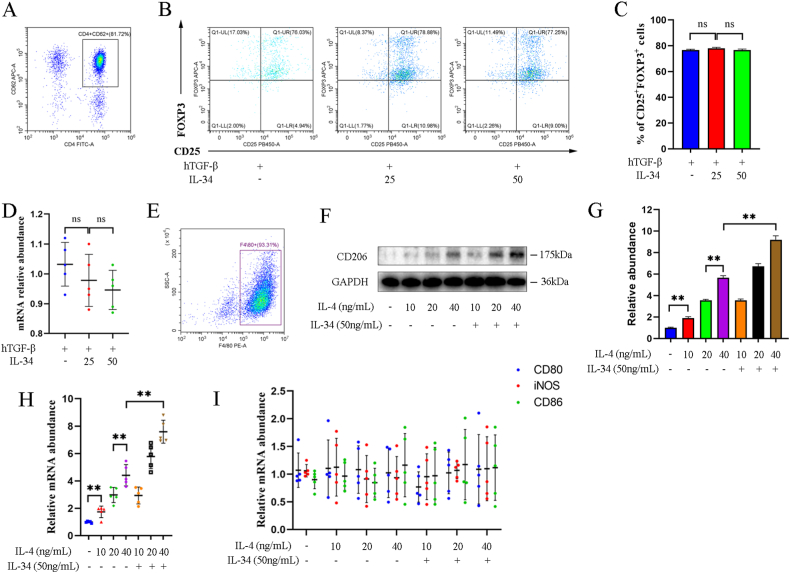
IL-34 promoted macrophage M2 polarization but not the differentiation of regulatory T cells (Tregs) *in vitro*. (A) Representative plot of CD4^+^CD62^+^ naïve T cells sorted from spleen with CD4 microbeads. (B) Representative plots of CD25^+^Foxp3^+^ Tregs gated from CD4^+^ population. (C) Graphic presentation showing the percentages of CD25^+^Foxp3^+^ Tregs. (D) Quantitative real-time polymerase chain reaction (qRT-PCR) analysis showing the mRNA abundance of Foxp3 in T cells. (E) Representative plot of F4/80+ macrophages analyzed by flow cytometry. (F, G) Western blot assay (F) and quantitative analysis (G) showing the protein abundance of CD206 in BMDMs treated with or without IL-34 (50 ng/ml). (H) qRT-PCR analysis of CD206 mRNA expression in BMDMs treated with or without IL-34 (50 ng/ml). (I) qRT-PCR analysis of CD80, iNOS and CD86 mRNA expression in BMDMs treated with or without IL-34 (50 ng/ml). *P < 0.05, **P < 0.01. Data are presented as means ± SD.

Naïve T cells sorted from spleen with CD4 microbeads were cultured *in vitro* (Fig. 6A). Flow cytometry showed no significant difference in Tregs conversion among groups, suggesting that IL-34 could not stimulate the differentiation of naïve T cells to Tregs (*P* > 0.05) (Fig. 6B and C). There was no significant difference in the mRNA abundance of Foxp3 (*P* > 0.05) (Fig. 6D). In addition, BMDMs were cultured to detect the effect of IL-34 on macrophage (Fig. 6E). The protein and mRNA abundance of CD206 were significantly increased in a dependent manner of IL-4 concentration, while the addition of IL-34 increased the protein and mRNA abundance of CD206 (*P* < 0.01) (Fig. 6F–H, S2B). However, there was no significant difference in the mRNA abundance of CD80, iNOS and CD86 expressed by M1 macrophages (*P* > 0.05) (Fig. 6I). These results indicated that IL-34 promoted macrophage M2 polarization but not the differentiation of Tregs *in vitro*.

## 4 Discussion

In our study, allogeneic mice renal transplant TCMR model was successfully established and validated. Based on it, the expression of IL-34 was decreased in TCMR allografts, while overexpression of IL-34 increased the expression of Tregs and M2 macrophages in the spleens and allografts of TMCR mice. Furthermore, IL-34 could promote macrophage M2 polarization but fail to induce differentiation of naïve T cells into Tregs *in vitro*.

When it comes to autoimmune diseases, IL-34 is associated with rheumatoid arthritis, Sjogren's syndrome and lupus nephritis [[14], [15], [16]]. Moreover, IL-34 alleviates rejection in both heart and liver grafts [9,17]. However, there have been no studies on the association of IL-34 with acute TCMR of mouse renal transplantation models. Immunohistochemical staining showed that IL-34 was clearly expressed in renal tubules of isografts and allografts, which was consistent with previous studies [18]. Based on this finding, we further investigate the role of IL-34 in TCMR in renal transplantation. We found that serum IL-34 were significantly higher in the TCMR group than in the SYN group, which was inconsistent with its expression in the TCMR kidneys. Serum IL-34 has been reported upregulated in mouse kidney transplantation models [19] and acute rejection of human liver transplantation [20], which is consistent with our results. As a cytokine of a variety of lymphocytes, IL-34 may cause circulatory rejection after renal transplantation due to graft stimulation, generous lymphocytes activated and systemic reactivity increased. However, as foreign antigens, local immune response of grafts was quite limited, which made IL-34 didn't show synchronization. According to the latest Banff criteria, IL-34 overexpression alleviated interstitial inflammation, tubulitis and small arteritis in allografts. Altogether, these data suggest that IL-34 has immunosuppressive properties to ameliorate TCMR of allografts.

CSF-1R, the primary receptor for IL-34, is widely present in monocytes and tissue macrophages. M2 macrophages are derived from IL-4 or IL-13-induced M0 macrophages secreted by innate and adaptive immune cells [21]. Activated M2 macrophages have anti-inflammatory effects, reducing inflammation and promoting tissue repair. Zhao et al. suggested that IL-34 inhibits acute rejection after rat liver transplantation by promoting M2 polarization [17]. It has been reported that Tregs produce IL-34 in rat heart transplantation models and human cell lines, which further induces the expansion of Tregs by promoting the differentiation of M2-type macrophages [9]. Consistent with the previous reported, infiltration of M2 macrophages increased in AAV-IL-34 allografts. Moreover, we found that rather than CD80, iNOS and CD86, CD206 abundance was increased by IL-34 treatment *in vitro*, which verified that IL-34 had a significant promoting effect on the polarization of macrophages from M0 to M2. Therefore, IL-34 overexpression might affect Tregs by promoting M2 polarization, which ultimately alleviates TCMR in allografts.

As a subpopulation of T cells, Tregs inhibit immune activation and limit peripheral self-immunity. It was reported that Tregs significantly ameliorated the loss of grafts [22]. In humans, Tregs have also been studied for clinical use as a therapeutic modality [23,24]. We found that efficient Tregs were generated following IL-34 treatment in TCMR of allografts and that Tregs could induce immune tolerance. In addition, the proportion of CD4-positive helper T cells and CD8-positive toxic T cells in the spleens showed no difference after AAV-IL-34 administration, whereas the proportion of Tregs in the spleens of AAV-IL-34 allografts was significantly higher than that in the controls, confirming the correlation between IL-34 overexpression and the increasing number of Tregs. However, there was no obvious difference in the conversion rate between the groups *in vitro*. Séverine et al. suggested that IL-34 prolongs survival in humanized acute GVHD models by increasing the expansion of Tregs rather than stimulating the transformation of naïve T cells to Tregs [25], which partly explained why IL-34 failed to induce Treg differentiation directly *in vitro*. These results highlighted that IL-34 exhibited immunosuppressive properties by inducing upregulation of Tregs.

Indeed, we have noticed some limitations that impede obtaining strong evidence of IL-34 in TCMR of allografts. Firstly, specific mechanism of IL-34 on Tregs and M2 macrophages is not clear. Then, the presence of IL-34 in other types of rejection model needs to be considered. Finally, clinical specimens were difficult to obtain to verify our findings.

## 5 Conclusion

In summary, we provided what we believe to be the first proof that IL-34 had potential as a therapy in TCMR. Overexpression of IL-34 was observed to attenuate the progression of TCMR episodes in kidney allografts by inducing the differentiation of Tregs and polarization of M2 macrophages in the spleens and allografts of TMCR mice. Importantly, IL-34 could promote macrophage M2 polarization but fail to induce differentiation of naïve T cells into Tregs *in vitro*. IL-34 may become a potential therapeutic strategy for TCMR of allografts.

## Data availability statement

All of data were presented in the main paper. The data that support the findings of this study are available from https://data.mendeley.com/datasets/bmgx9pnrrg/3. All authors take responsibility for the integrity of the data and the accuracy of the data analysis.

## Funding

ZJ Wang, M Gu, RY Tan.

## CRediT authorship contribution statement

**Bin Ni:** Writing – review & editing, Writing – original draft, Visualization, Validation, Methodology, Investigation, Data curation. **Dongliang Zhang:** Writing – review & editing, Writing – original draft, Software, Resources, Methodology, Formal analysis. **Hai Zhou:** Validation, Supervision, Resources, Investigation, Formal analysis, Data curation. **Ming Zheng:** Validation, Investigation, Formal analysis, Data curation. **Zijie Wang:** Resources, Project administration, Methodology, Investigation. **Jun Tao:** Validation, Supervision, Resources, Methodology. **Zhijian Han:** Validation, Supervision, Resources. **Xiaobin Ju:** Validation, Supervision, Methodology. **Ruoyun Tan:** Writing – review & editing, Visualization, Validation, Resources, Project administration, Methodology, Funding acquisition. **Min Gu:** Writing – review & editing, Visualization, Validation, Supervision, Resources, Project administration, Methodology, Investigation, Funding acquisition.

## Declaration of competing interest

The authors declare that they have no known competing financial interests or personal relationships that could have appeared to influence the work reported in this paper.
