# Supplementary material for: IL-34 attenuates acute T cell-mediated rejection following renal transplantation by upregulating M2 macrophages polarization
Source: Heliyon. 2024 Jan 3;10(1):e24028. doi: 10.1016/j.heliyon.2024.e24028 (PMC10789621; doi:10.1016/j.heliyon.2024.e24028)
Supplement: Multimedia component 1 [file mmc1.docx]

**Supplementary materials**

S1: IL-34 expression in recipients increased after the intervention of adeno-associated virus mediated overexpression of IL-34.


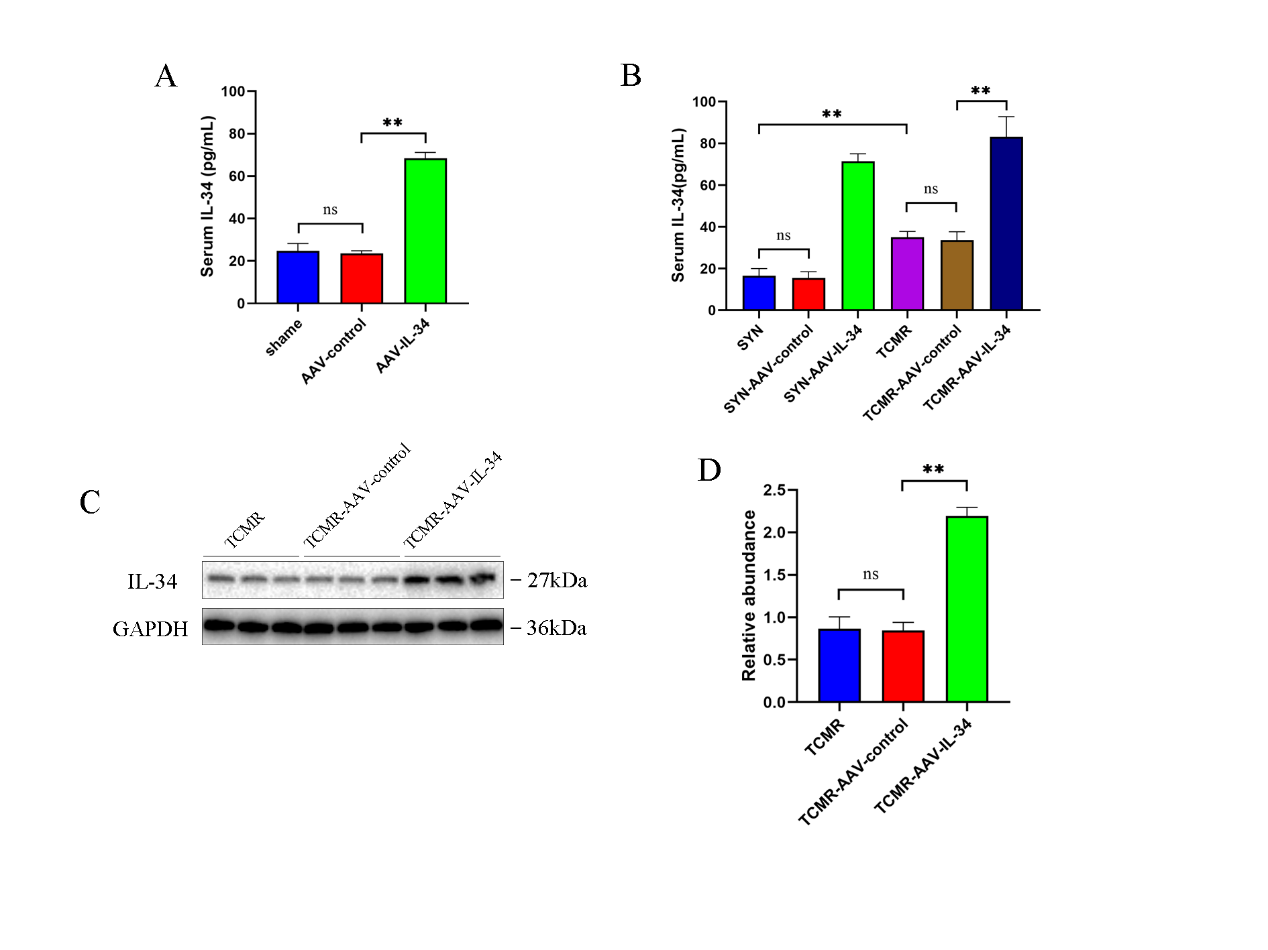


(A) Serum IL-34 concentration was determined by ELISA assays at day 21 after injection of AAV. (B) Serum IL-34 concentration was determined by ELISA assays at day 7 after renal transplantation. (C) Western blot and (D) quantification showing the upregulation of IL-34 in the liver of mice at day 7 after renal transplantation. *P<0.05, **P<0.01. Data are presented as means ± SD.

S2: Original images of WB assay.


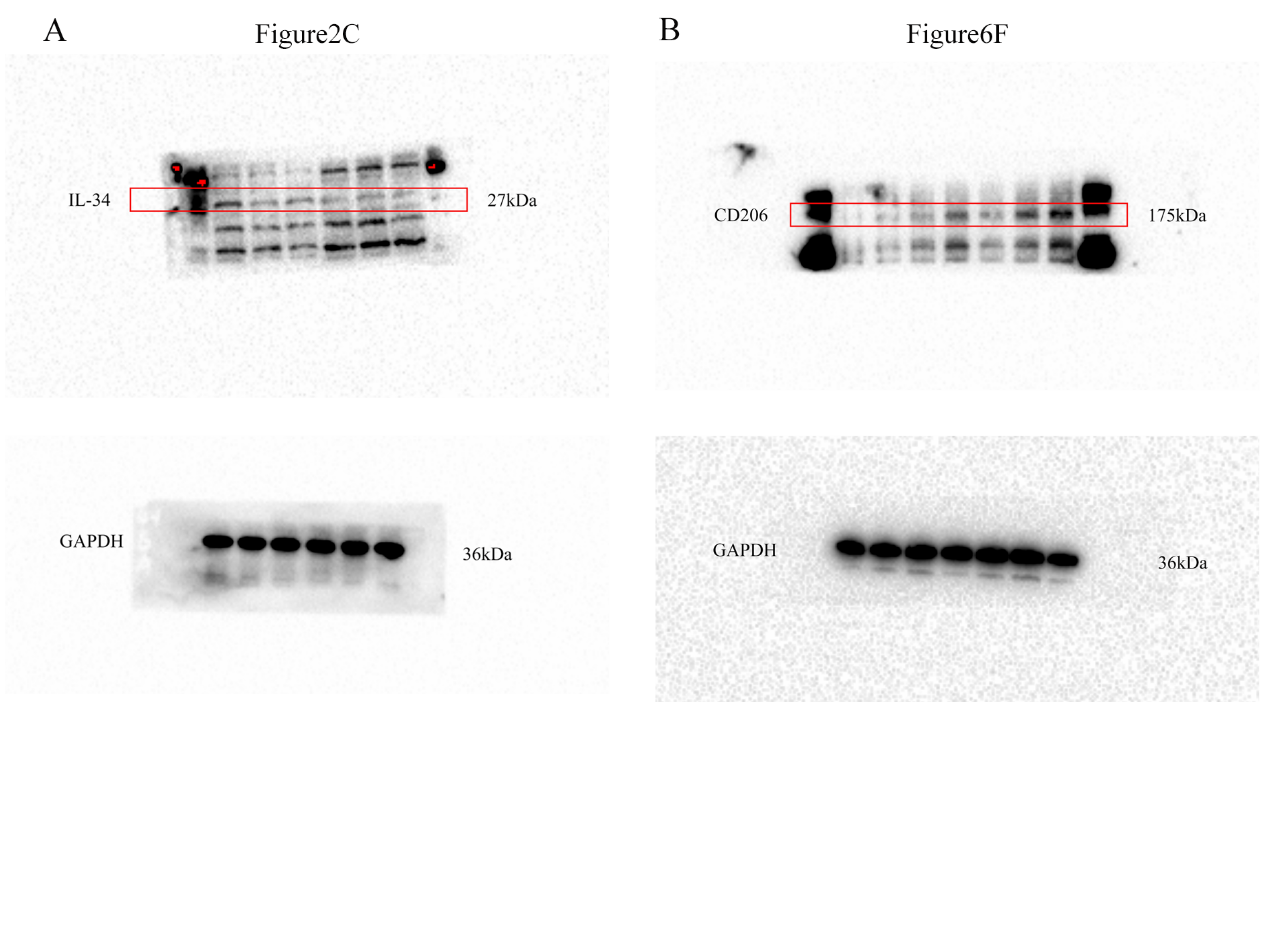


(A) Original images of WB assay of Figure 2C. (B) Original images of WB assay of Figure 6F.
